# Evidence of Molecular Evolution Driven by Recombination Events Influencing Tropism in a Novel Human Adenovirus that Causes Epidemic Keratoconjunctivitis

**DOI:** 10.1371/journal.pone.0005635

**Published:** 2009-06-03

**Authors:** Michael P. Walsh, Ashish Chintakuntlawar, Christopher M. Robinson, Ijad Madisch, Balázs Harrach, Nolan R. Hudson, David Schnurr, Albert Heim, James Chodosh, Donald Seto, Morris S. Jones

**Affiliations:** 1 Department of Bioinformatics and Computational Biology, George Mason University, Manassas, Virginia, United States of America; 2 Howe Laboratory, Massachusetts Eye and Ear Infirmary, Harvard Medical School, Boston, Massachusetts, United States of America; 3 Insitut für Virologie, Medizinische Hochschule, Hannover, Germany; 4 Veterinary Medical Research Institute, Hungarian Academy of Sciences, Budapest, Hungary; 5 Clinical Investigation Facility, David Grant USAF Medical Center, Travis AFB, California, United States of America; 6 Viral and Rickettsial Disease Laboratory, California Department of Public Health, Richmond, California, United States of America; University of Pretoria, South Africa

## Abstract

In 2005, a human adenovirus strain (formerly known as HAdV-D22/H8 but renamed here HAdV-D53) was isolated from an outbreak of epidemic keratoconjunctititis (EKC), a disease that is usually caused by HAdV-D8, -D19, or -D37, not HAdV-D22. To date, a complete change of tropism compared to the prototype has never been observed, although apparent recombinant strains of other viruses from species *Human adenovirus D* (HAdV-D) have been described. The complete genome of HAdV-D53 was sequenced to elucidate recombination events that lead to the emergence of a viable and highly virulent virus with a modified tropism. Bioinformatic and phylogenetic analyses of this genome demonstrate that this adenovirus is a recombinant of HAdV-D8 (including the fiber gene encoding the primary cellular receptor binding site), HAdV-D22, (the ε determinant of the hexon gene), HAdV-D37 (including the penton base gene encoding the secondary cellular receptor binding site), and at least one unknown or unsequenced HAdV-D strain. Bootscanning analysis of the complete genomic sequence of this novel adenovirus, which we have re-named HAdV-D53, indicated at least five recombination events between the aforementioned adenoviruses. Intrahexon recombination sites perfectly framed the ε neutralization determinant that was almost identical to the HAdV-D22 prototype. Additional bootscan analysis of all HAdV-D hexon genes revealed recombinations in identical locations in several other adenoviruses. In addition, HAdV-D53 but not HAdV-D22 induced corneal inflammation in a mouse model. Serological analysis confirmed previous results and demonstrated that HAdV-D53 has a neutralization profile representative of the ε determinant of its hexon (HAdV-D22) and the fiber (HAdV-D8) proteins. Our recombinant hexon sequence is almost identical to the hexon sequences of the HAdV-D strain causing EKC outbreaks in Japan, suggesting that HAdV-D53 is pandemic as an emerging EKC agent. This documents the first genomic, bioinformatic, and biological descriptions of the molecular evolution events engendering an emerging pathogenic adenovirus.

## Introduction

Epidemic keratoconjunctivitis (EKC), characterized by inflammation of the conjunctiva and cornea, produces a sudden onset of acute follicular conjunctivitis and stromal keratitis and is a worldwide problem causing significant and sometime lasting morbidity [1]. Human adenoviruses (HAdVs) HAdV-D8, -D19, and -D37 are the most common pathogens causing EKC [1].

Adenoviruses were first isolated from civilians and military trainees who had respiratory disease in the early 1950s [2], [3]. They were the first respiratory viruses to be isolated and characterized. Epidemiological studies confirmed that adenoviruses are the cause of acute febrile respiratory disease among military recruits [4], [5] and have been persistent in the global population. Since then, 52 human adenovirus (HAdV) genotypes have been characterized and classified according to their immunochemical properties, nucleic acid similarities, hexon and fiber protein characteristics, biological properties, and phylogenetic analysis, and placed in the genus *Mastadenovirus* [6], [7]. These 52 adenovirus genotypes that infect humans are classified into seven species (*Human adenovirus A* to *G*) [6], [8] and are known to cause a range of diseases specific to the tropisms of the viruses: keratoconjunctivitis (HAdV-D8, HAdV-D19, and HAdV-D37) [9], [10], gastroenteritis (HAdV-A31, HAdV-F40, HAdV-F41, and HAdV-G52) [6], acute respiratory disease (HAdV-B3, HAdV-E4, HAdV-B7, HAdV-B14, and HAdV-B21) [5], and perhaps obesity (HAdV-D36) [11].

Adenoviruses have linear double-stranded DNA genomes that generally range from 26 to 45 kb and are encapsidated in an icosahedral protein shell that ranges from 70 to 100 nm [8]. The primary components of the protein shell are the hexon, penton base, and fiber proteins. Through genome sequence analysis, it has been demonstrated that the genomes of all human adenoviruses have similar genetic organization [12], [13], [14].

In the past, human adenovirus serotype and species classification were defined by reactivity of outer coat proteins to discriminating antibodies (e.g., immunochemistry/virus neutralization) as well as by other biological properties (e.g. oncogenic potential, hemagglutination properties). Today, given the availability of DNA sequencing and analysis technology, phylogenetics (based on comparative nucleic acid and amino acid sequence analysis of informative viral proteins or/and their genes, as well as analysis of genomic organization) is a highly quantitative, cost-effective, expedient method and the preferred and reliable method for classifying adenoviruses. It is a preferred and reliable method for demonstrating how viruses are related through molecular evolution as it provides and relies on the primary sequence data [15], [16], [17], [18].

In this study we sought to characterize a unique intermediate recombinant HAdV isolate, at the molecular level. This novel strain was isolated from a patient who, along with eleven other patients, presented with highly contagious EKC outbreak in Germany was described [19]. Since HAdV-D22 was never associated with EKC, we performed whole genome sequencing, complemented with bioinformatics, including phylogenetic and in silico proteome analysis, as well as *in vivo* studies in a mouse model to characterize this unique recombinant virus. To reflect this novel and different genome and because of the multiple recombination events and several unique sequence segments in the genome of this virus, we renamed this virus HAdV-D53.

## Results

### Amplification and sequencing of the new adenovirus

Initial and partial sequencing of HAdV-D53 (previously HAdV-D22/H8) demonstrated that portions of the penton and fiber genes were similar to HAdV-D37 and HAdV-D8, respectively [19]; thus suggesting that this disease causing virus was the result of recombination. To understand clearly the genetic characteristics and the nature of HAdV-D53, the entire genome has been sequenced and analyzed.

### Physical features of new adenovirus genome

**Figure 1:**
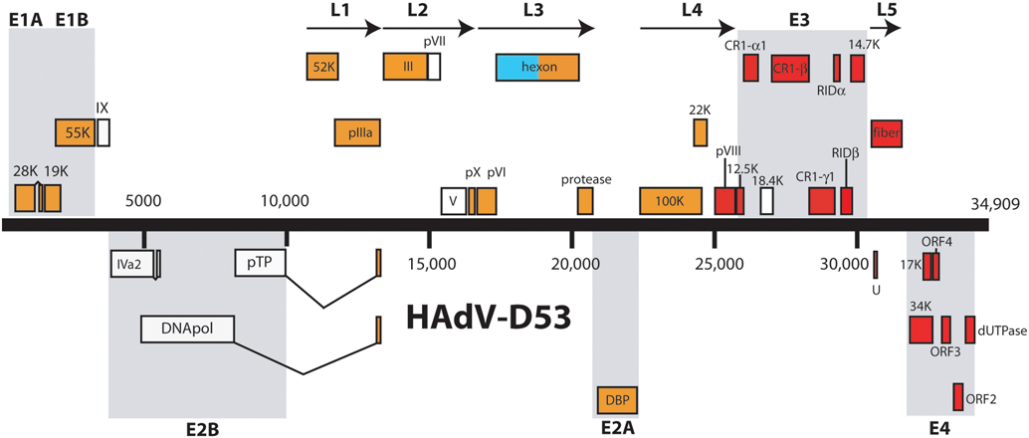
Genome organization of HAdV-D53. Genome is represented by a central black horizontal line marked at 5-kbp intervals. Protein-encoding regions are shown as boxes. Boxes above the black line represent open reading frames (ORFs) that are encoded on the forward (or upper) strand. Boxes underneath the black line represent ORFs that are encoded on the reverse (or lower) strand. The colors of the boxes correspond to which adenovirus the protein is most likely descended from: red – HAdV-D8, aqua – HAdV-22, orange – HAdV-D37, white – dissimilar to all known adenoviruses.

The genome length of HAdV-D53 is 34,909 base pairs, with a base composition of 23% A, 20.8% T, 28.2% G, 28% C and the GC content was 56.2%. The GC content is consistent with members of species *Human adenovirus D* (HAdV-D) (57.0% mean). The organization of the 36 open reading frames (ORF's) that were found had a genome organization similar to other mastadenoviruses (Fig. 1). The inverted terminal repeat (ITR) sequences for HAdV-D53 were determined to be 212 bp in length.

The nucleotide and amino acid identities for selected genes in the genome of HAdV-D53 to its nearest relatives are shown in Tables 1 and 2, respectively. Interestingly, the pVII and protein V genes were dissimilar to homologous genes in any adenovirus species with 83 and 87% nucleotide identity, respectively to HAdV-D37 (Table 1), the nearest relative in that region of the HAdV-D53 genome. For pVII, the low nucleotide identity is partially due to a 99 bp deletion, resulting in a 33 amino acid deletion of the predicted pVII protein. When compared to HAdV-D37, the protein V gene contains 2 deletions. The first deletion is 18 bp and the second is 93 bp.

**Table 1:** Percent identities of the nucleotide coding sequences of selected HAdV-D53 proteins and their homologs^a^.

	HAdV-D8p^b^	HAdV-D19	HAdV-D22	HAdV-D37	HAdV-D48	HAdV-D49
**E1B 19K**	94.9	99.3	99	**99.5**	97.8	97.8
**E1B 55K**	96	99.2	98.3	**99.3**	97.4	96.9
**IX**	95.1	96.1	98.5	96.1	97.1	97.1
**IVa2**	96.7	98	97.4	98	98.3	91.3
**DNA polymerase**	95.2	98.1	97.6	98	98	98
**pTP**	93.3	97.6	96.8	97.9	96.4	96.8
**52K**	95.7	98	98.1	**100**	97.9	98.3
**penton base**	89.2	91.2	92.4	**100**	90.5	90.1
**pVII**	79.2	80.4	80.4	83.2	80	80.1
**V**	85.2	87	87.5	87.2	87.7	87.3
**pX**	95.1	100	100	**100**	98.7	99.6
**hexon**	89.4	90.2	***98.4***	***90.5***	90.6	90.4
**protease**	95.4	96	95.4	**99.8**	96.5	96.5
**22K**	89.9	100	98.8	**100**	99.3	99
**pVIII**	95.6	98.4	98.5	98.4	98.3	97.7
**12.2K**	93.2	96	97.5	96	96.9	96.9
**CR1-α**	52.5	80.5	97	80.5	75.4	77.6
**18.4K**	95.2	91.1	98.1	91.1	96.2	94.1
**CR1-β**	**100**	74.5	85.1	74.5	64.5	58
**CR1-γ**	86.4	75.3	Nd^c^	75.3	75.1	80.5
**RID-α**	93.5	94.2	94.2	94.2	98.2	98.2
**RID-β**	90.6	87.4	93.2	87.4	94.2	99.2
**14.7K**	96.7	95.2	97.2	95.2	96.7	97.7
**fiber**	**100**	75.1	67.6	75	69	67.6
**dTPase**	**100**	48.4	88.9	48.4	85.1	85

Standard nomenclature has been applied so that orthologs have the same name (Davison et al., 2003). Numbers in bold reflect the proposed origin. Italics note the gene with supposed double origin.

a Percent identities and similarities were determined by global alignment using the EMBOSS needle program with a gap penalty of 10.0 and a gap extension penalty of 5.0.

b Not present in the genome.

c Prototype HAdV-D8 strain is Trim isolate – ATCC VR-1604.

**Table 2:** Percent identities of selected amino acid sequences of HAdV-D53 proteins and their homologs.

	HAdV-D8	HAdV-D8p	HAdV-D19	HAdV-D22	HAdV-D37	HAdV-D48	HAdV-D49
**E1B19K**		92	99	99	**100**	97	97
**E1B55K**		96	99	98	**99**	97	96
**IX**		95	97	99	97	97	98
**IVa2**		97	99	99	**99**	99	98
**DNA polymerase**		96	98	98	98	99	98
**pTP**		94	97	97	98	97	97
**52K**		96	99	99	**100**	98	98
**penton base**		89	91	92	**100**	89	89
**pVII**		79	80	80	81	79	80
**V**		84	87	87	87	87	87
**pX**		100	100	100	**100**	100	100
**hexon**		92	90	99	90	92	90
**protease**		97	100	99	**100**	100	100
**DBP**		96	99	98	**100**	97	97
**22K**		81	100	99	**100**	99	98
**pVIII**	**100**	97	98	99	98	98	98
**12.5K**	**100**	95	95	99	95	98	98
**CR1-α**	**99**	54	73	94	73	67	71
**18.4K**	97	95	91	97	91	94	92
**CR1-β**	97	**100**	74	81	74	48	44
**CR1-γ**	**100**	**81**	62	65	62	65	73
**RID-α**	**100**	94	29	96	96	100	100
**RID-β**	**100**	89	92	93	92	89	97
**14.7K**	**100**	96	94	97	94	97	98
**fiber**	**100**	**100**	74	62	74	63	59
**dUTPase**		**100**	92	87	92	84	82

Numbers in bold reflect the proposed origin. Italics note the gene with supposed double origin.

### Genomic recombination analysis

**Figure 2:**
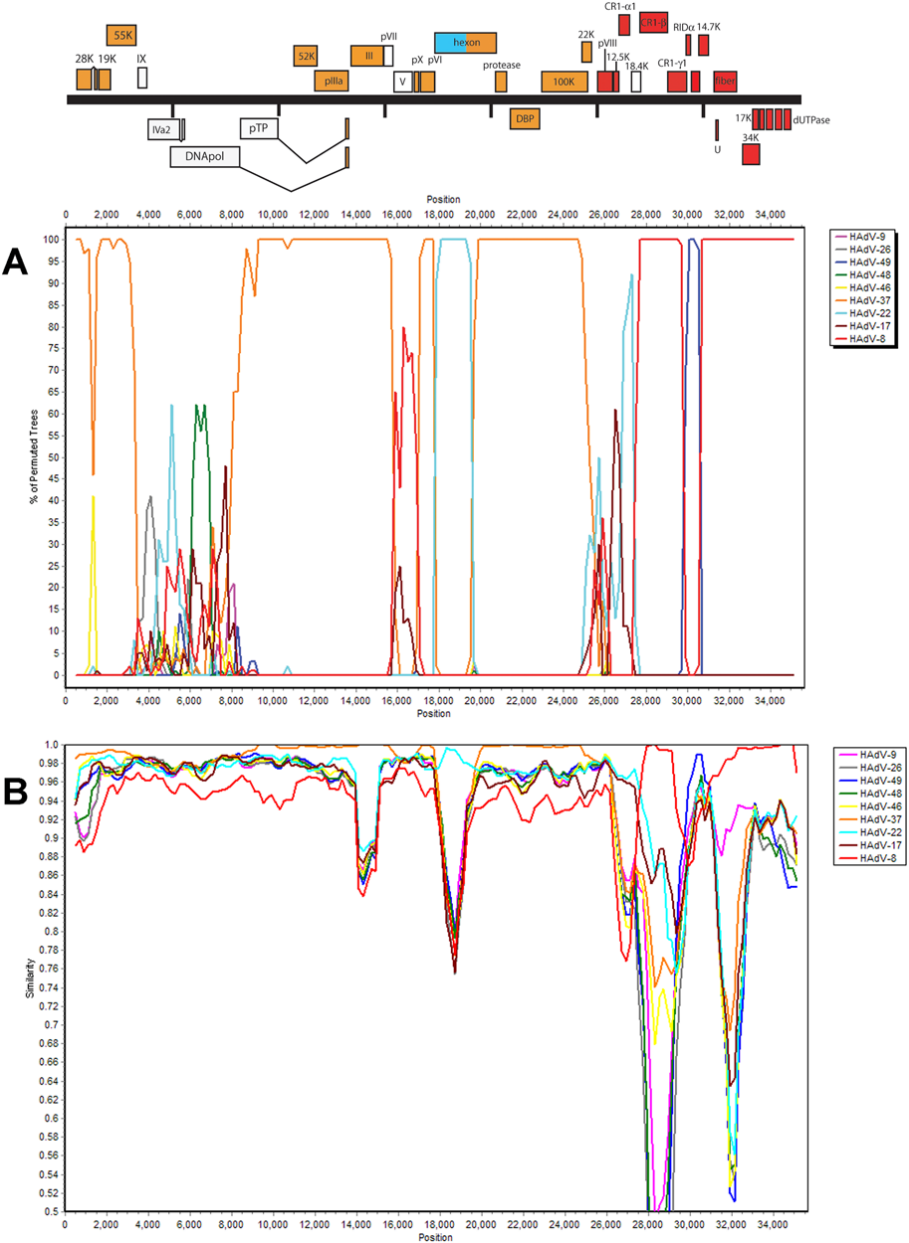
Whole genome (A) bootscan and (B) simplot of HAdV-D53 compared to fully sequenced HAdV-D genomes.

To determine if recombination occurred within the HAdV-D53 genome, several software tools were applied. A bootscanning program [20] was used to determine the relationship of HAdV-D53 to all of the fully sequenced HAdV-D genotypes. According to the alignments, several regions indicated recombination events; nucleotides (as genome coordinates) 1–1000, 1500–3250 (E1A, E1B, 55K), 8500–15,750 (52K, pIIIa, penton base), 17,000–17,750, (pX, pVI) and 19,500–25,000 (second half of hexon, protease, DBP, 100K, 22K) showed a strong relationship to HAdV-D37; nucleotides 17,750–19,500 (first half of hexon) showed a strong relationship to HAdV-D22; nucleotides 27,375–29,750 (CR1-β) and 30,500–34,909 (14.7K, fiber, E4 ORFs) showed a strong relationship to HAdV-D8 (Fig. 2). Although the bootscan analysis showed that nucleotides 29750–30,500 have a strong relationship to HAdV-D49, we believe that this region comes from an unsequenced HAdV-D8 strain, because that region (RIDβ) in a partially sequenced HAdV-D8 strain has 100% amino acid identity to the Hiroshima HAdV-D8 isolate (Table 2). These relationships were confirmed by comparison with nucleotide identity in Table 1, as well as BLASTP similarity analysis of the proteins (Table 2). In contrast, nucleotides 1000–1500, 3250–8500, 15,750–17,000, and 25,000–27,375 showed slightly lower similarity to several known adenoviruses, suggesting that this region of species HAdV-D adenoviruses are both well conserved and so far unique for the studied strain. Thus, bootscan analysis of the HAdV-D53 genome shows evidence of multiple recombination events.

### Hexon recombination analysis

**Figure 3:**
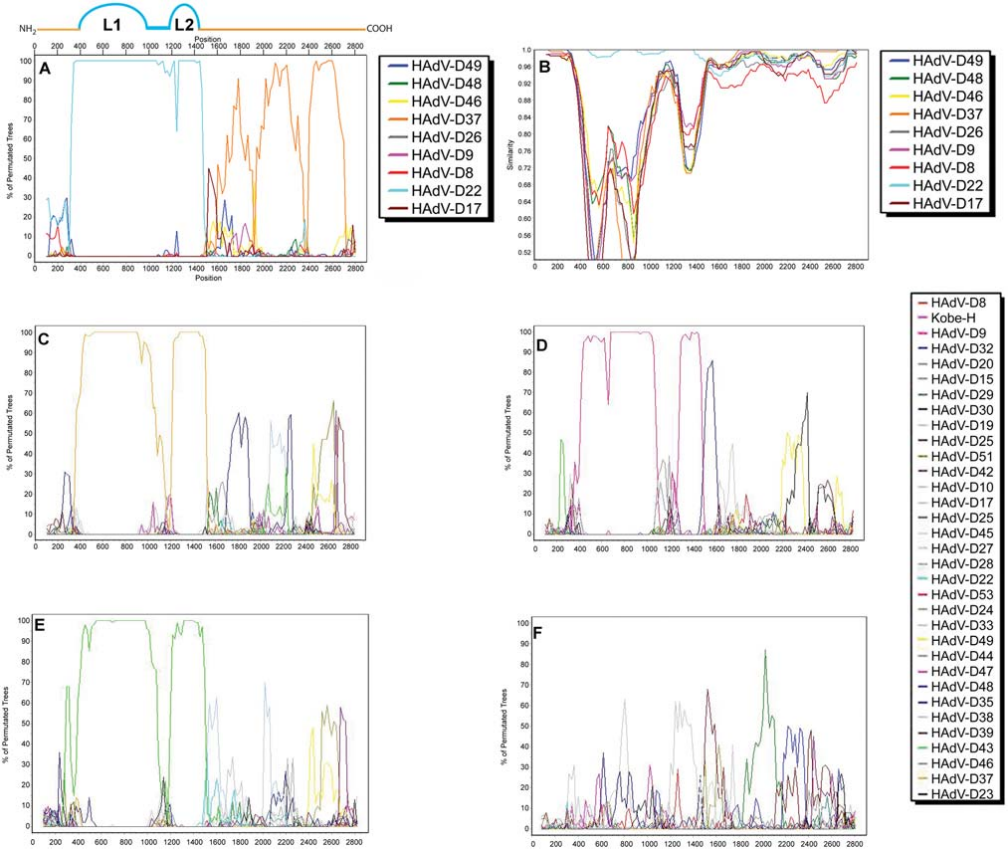
Bootscan of HAdV-D species hexon genes demonstrating recombination events. Comparison of HAdV-D53 by (A) bootscan and (B) simplot with the HAdV-D types which have a fully sequenced genome. (C) HAdV-D13, (D) HAdV-D32, (E) HAdV-D39, and (F) HAdV-D49 were compared to all hexon genes in species HAdV-D by bootscan analysis.

The results of our whole genome bootscan indicated that a recombination event occurred inside the hexon gene. The hexon contains loops 1 (L1) and 2 (L2), which are the most important determinants of neutralization via antibodies as well as immune escape. Since L1 and L2 are the most relevant for serotyping, we performed bootscan analysis to pinpoint where the recombination events occurred in the hexon gene of HAdV-D53. The results of the bootscan analysis shown in Figure 3A and 3B reveal that a recombination event occurred between nucleotide 380 and 1400–1620 which are the amino terminus of L1 and the conserved C terminus of the highly variable L2, respectively (Table 3). Thus, the complete neutralization epitope ε, which is nearly identical to the sequenced HAdV-D22, is framed by non HAdV-D22 sequences in the recombinant strain HAdV-D53.

**Table 3:** An excerpt from the plot values of a Simplot Bootscan of the HAdV-22D and HAdV-D37 hexons.

Center Pos	HAdV-D22 hexon	HAdV-D37 hexon
1320	**100**	0
1340	**100**	0
1360	**100**	0
1380	**100**	0
1400	**100**	0
1420	**100**	0
1440	**100**	0
1460	80	0
1480	18	1
1500	1	12
1520	0	7
1540	0	7
1560	0	3
1580	0	2
1600	0	46
1620	0	51
1640	0	45
1660	0	51
1680	0	60
1700	0	57
1720	0	50
1740	0	80

Based on the nucleotide identity of HAdV-D53 to other adenoviruses, we believe that the previous name HAdV-D22/H8 is not appropriate due to the fact that the fully sequenced genome and the bioinformatic analyses demonstrate that HAdV-D53 is the product of multiple recombinations of known and perhaps undiscovered and/or yet unsequenced adenoviruses. Taken together, we propose the name “HAdV-D53” for this novel recombinant adenovirus, reflecting its genome divergence from other human adenoviruses. We also believe this “genome type” designation is more appropriate in light of the current and future DNA sequencing and analysis technology, superseding the importance of the previous classifications based on serology (e.g., serotypes).

### Hexon recombination is common in species *Human adenovirus D*

To determine whether or not this phenomenon was common in other adenoviruses, the available hexon genes of all HAdV-D genotypes were cross-examined. Recombination events at similar nucleotide locations of the hexon gene in HAdV-D13, HAdV-D32, and HAdV-D39 (Fig. 3C–E) were found. The HAdV-D13 recombination is especially interesting regarding the present study, as HAdV-D13 acquired L1 and L2 from HAdV-D37 and the same region in HAdV-D53 was presumably exchanged for L1 and L2 of HAdV-D22. To demonstrate the validity of our recombination predictions, we included the bootscan analysis of the HAdV-D49 hexon gene, which does not show evidence of any recombination events (Fig. 3F). Taken together, these data suggest that adenoviruses in HAdV-D species are susceptible to recombination events at the amino terminus of L1 and the carboxy terminus of L2 of the hexon gene; implicating a mechanism which allows adenoviruses to switch neutralization epitopes.

### *In vivo* HAdV-D53 induced keratitis

**Figure 4:**
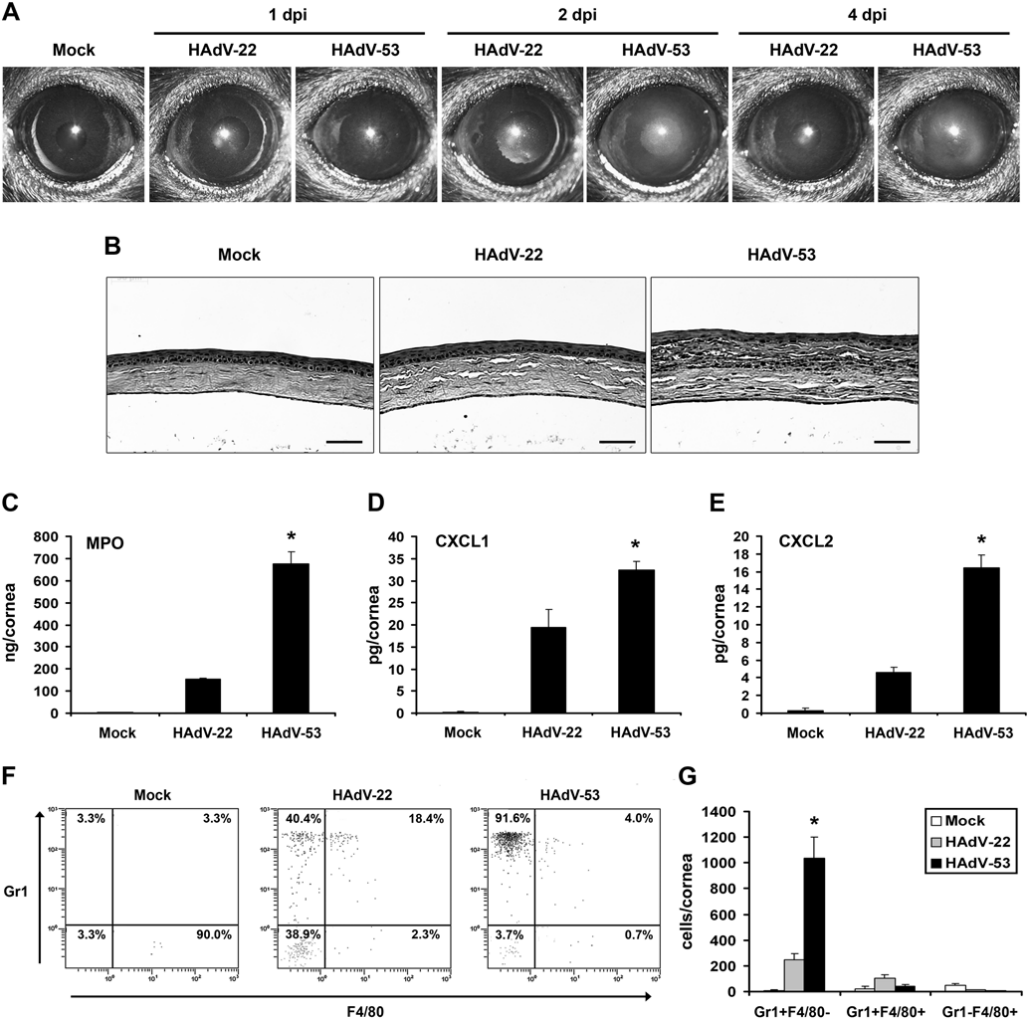
HAdV-D53 induces keratitis. (A) Clinical appearance of HAdV-D53 keratitis. Virus-free buffer (mock), 10^4^ TCID of HAdV-D22, or HAdV-D53 was injected in the corneal stroma of C57BL/6 mice (n = 8 corneas/group). Corneas were examined under a surgical microscope up to 4 days post-infection. One representative picture from each group is shown at the indicated time points. (B) Histopathology of HAdV-D53 keratitis. Representative histopathological sections at 4 days post-infection of mouse corneas injected with buffer, HAdV-D22, or HAdV-D53 are shown (hematoxylin and eosin stain; scale bar = 50 µ). (C) Myeloperoxidase (MPO) expression in HAdV-D53 keratitis. Mock, HAdV-D22, and HAdV-D53 infected corneas were analyzed by ELISA at 24 hours post-infection for the expression of myeloperoxidase enzyme. (D, E) Chemokine expression in HAdV-D53 keratitis. Expression of CXCL1 (D) and CXCL2 (E) in mock, HAdV-D22, and HAdV-D53 infected corneas were analyzed by ELISA at 16 hpi. Data is mean±SEM from three individual experiments (n = 9 corneas/group). (F, G) Phenotypic analysis of inflammatory cells in HAdV-D53 keratitis. Mock, HAdV-D22, and HAdV-D53 infected corneas at 24 hours post-infection were homogenized and single cell preparations were stained with anti-CD45, anti-Gr1, and anti-F4/80 antibodies. Cells were gated on CD45-positive staining. (F) Representative dot plots or (G) quantification of three separate experiments is shown for each group (mean cells/cornea±SEM, n = 9 corneas/group). In all experiments statistical significance is denoted by *, P<.05 as determined by ANOVA with Scheffe's multiple comparison test.

Since HAdV-D53 was isolated from a patient with EKC and appeared to be corneotropic [19], we tested its ability to induce corneal innate immune responses in a previously described mouse model of adenovirus keratitis, in which EKC viruses induce a keratitis similar to human EKC, but without viral replication [21]. HAdV-D53 infection induced a clinically evident keratitis (corneal opacity) as early as 1 day post-infection (dpi) that peaked by 3–4 dpi (Fig. 4A). In contrast, mock and HAdV-D22 injection did not induce corneal opacity at any time post-infection. Neither virus replicated in the mouse cornea (data not shown). Hematoxylin and eosin staining of corneal cross sections at 4 dpi with HAdV-D53 showed thinning of the epithelial cell layer, stromal edema, and infiltration by leukocytes (Fig. 4B). In contrast, HAdV-D22 infection induced only modest cellular infiltration. We next assessed corneal myeloperoxidase (MPO) levels after infection as a measure of the presence of infiltrating neutrophils and monocytes [22], [23]. HAdV-D53 infection induced significantly higher levels of MPO when compared to HAdV-D22 and mock infected corneas (Fig. 4C). By flow cytometry, corneal infection with HAdV-D53 caused a significantly greater number of infiltrating neutrophils (Gr1+F4/80−) [24], [25], similar to previous studies with HAdV-D37 [21], than with HAdV-D22 infection. Inflammatory monocytes (Gr1+F4/80+) [26], [27] and resident macrophages (Gr1-F4/80+) [28] did not increase significantly after infection with either virus (Figs. 4F and G). Because neutrophils appeared by histology and flow cytometry to be the predominant infiltrating cell in HAdV-D53 keratitis, we also tested the expression of neutrophil chemokines CXCL1 and CXCL2 [29], [30]. Both CXCL1 (Fig. 4D) and CXCL2 (Fig. 4E) were expressed at significantly higher levels after infection with HAdV-D53 than with HAdV-D22.

### Phylogenetic analysis

**Figure 5:**
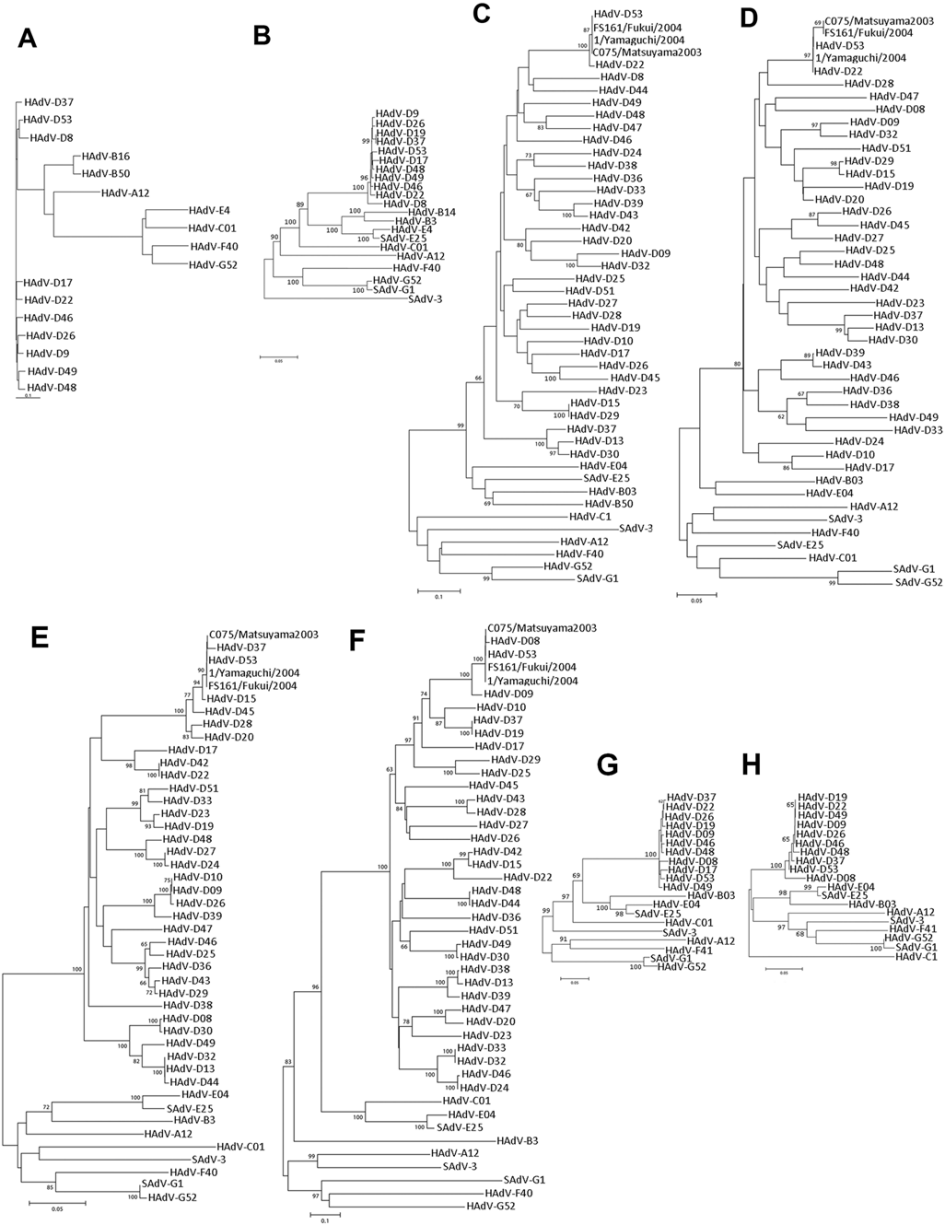
Phylogenetic analysis of HAdV-D53. Analysis of HAdV-D53 is based on the nucleic acid sequence of (A) complete genomes, as well as the predicted amino acid sequences of (B) polymerase, (C) L1 and (D) L2 of the hexon protein penton, (E) β-determinant, (F) γ-determinant, (G) pV and (H) pVII. Numbers denote human adenovirus serotypes. HAdV-D53 (in bold) shows the new isolate. The numbers close to the nodes represents bootstrap pseudoreplicates.

Detailed phylogenetic analysis of selected proteins, performed with nucleotide data and deduced amino acid sequences confirmed that HAdV-D53 was an unusual recombinant adenovirus. The tree topology of HAdV-D53 was different depending on which protein was tested. The penton base had the closest relationship to HAdV-D37, whereas the fiber gene was closest to HAdV-D8 (Fig. 5). Interestingly, DNA polymerase, protein V, and pVII genes did not cluster tightly with any other virus, which reflects their unique sequences. As expected the L1 and L2, which are responsible for the neutralization ε determinant, clustered with HAdV-D22. In L1, HAdV-D22 was 1.8% distant to HAdV-D53 and L2 was identical to HAdV-D53. In the β-determinant, HAdV-D53, supported by a strong bootstrap value (83%), clustered to HAdV-D37. Using sequence data that was available in GenBank, the hexon sequences of HAdV-D53 clustered tightly with the Japanese isolates 1/Yamaguchi/2004, C075/Matsuyama/2003, and FS161/Fukui/2004 suggesting that HAdV-D53 and the Japanese isolates represent different isolates of the same HAdV genotype.

### Viral neutralization

Since our sequence analysis shows that HAdV-D53 is genetically similar to HAdV-D8, -D22, and -D37, we wanted to determine its serum neutralization profile. Antisera to HAdV-D8 and HAdV-D22 neutralized HAdV-D53 at dilutions of 1∶128 and 1∶256, respectively. In contrast, antisera to HAdV-D37 was unable to neutralize HAdV-D53 at a dilution of equal to or less than 1∶8. These results confirm previous results and demonstrated that HAdV-D53 has a neutralization profile representative of its hexon and fiber proteins whereas the penton base did not contribute to neutralization.

## Discussion

The initial report of HAdV-D53 described that this novel, possibly emergent disease-causing, strain comprised a HAdV-D22 strain that had recombined with HAdV-D8 and HAdV-D37 [19]. Full genome sequencing of this isolate, HAdV-D53, and bioinformatic analysis have demonstrated that this genome is so different both from HAdV-D22 and all the other officially accepted serotypes that it must be seen as a novel human adenovirus which we have re-named HAdV-D53 based on its primary sequence data and analyses.

This genome is based on a highly probable homologous recombination between HAdV-D37 and HAdV-D8; however (probably after the initial recombination event), other parts of the genome have been replaced by genome parts from several known or unknown HAdVs. Altogether, we assume the occurrence of at least five major recombination events: (1) recombination of HAdV-D37 and HAdV-D8 (occurring between the end of the HAdV-D37 22K gene and the beginning of the HAdV-8 pVIII gene); (2) exchange with an unknown or unsequenced adenovirus from species HAdV-D from the beginning of the protein IX gene to the end of the pTP gene; (3) replacement of the pVII and protein V genes with the same genome fragment from an unknown or unsequenced adenovirus from species HAdV-D; (4) exchange of L1 and L2 between HAdV-D22 and the recombinant virus; (5) and replacement of the 18.4K gene from an unknown source.

Here we presented evidence that the neutralization epitope ε of HAdV-D53, highly homologous to HAdV-D22, was generated by two recombination events which brought about the complete exchange of L1 and L2. This phenomenon is apparent in three other adenoviruses from HAdV-D (HAdV-D13, -D32, and -D39), as well as HAdV-B16, which is a member of species HAdV-B [31]. This detailed analysis at the complete genome level demonstrates that recombination may be a common event within adenoviruses, especially in species HAdV-D, as a general mechanism driving molecular evolution and immunogenicity. The neutralization epitope is framed by highly conserved sequences, which are also used for generic detection of most HAdVs by PCR [32], [33], [34]. These conserved sequences allow homologous recombination when a cell is infected with two different adenovirus types. Our results demonstrate that within HAdV-D, the neutralization epitopes ε are exchangeable in nature leading to immune escape of a highly virulent and prevalent HAdV type. This resembles the antigenic shift mechanism of influenza A viruses which is caused by reassortment, a more efficient way of gene transfer.

To date, this is the first fully sequenced recombinant adenovirus to be associated with EKC. Bootscan analysis showed that several regions of HAdV-D53 (IVa2, DNA polymerase, pTP, pVII, V, and 18.4K) were dissimilar to any known adenovirus. These sequences are either from an undiscovered adenovirus or a known yet unsequenced HAdV-D isolate. Additional whole genome sequencing studies of adenoviruses will shed light on this important question.

In light of its association with EKC, it seems significant that experimental corneal infection with HAdV-D53 induced inflammation, while infection with HAdV-D22, a virus not associated with EKC but highly related to HAdV-D53, did not. Those areas of the genome unique to EKC-causing viruses represent likely sources of corneal tropism. Full genome sequencing, bioinformatics analysis, and genome wide comparisons between EKC and non-EKC inducing HAdV-D strains are beginning to yield clues to corneal tropism and pathogenesis [8], [9]. Further experiments recombining different adenovirus genes will determine which genes are crucial for EKC.

Early genotyping of HAdV-D53 by sequencing of the hexon (the major neutralization determinant) and other determinants (fiber and penton) gave results of a recombinant strain HAdV-D22/H8 [19]. Thus, HAdV-D53 fulfilled the hexon L1 and L2 criteria for typing as HAdV-D22 [18], with a fiber knob (hemagglutination determinant) sequence identical to HAdV-D8. In contrast to the classical concept of a recombinant strain, HAdV-D53 was cross reactive with a HAdV-D8 specific antiserum (Table 4). This confirms that some of the neutralization antibodies in the HAdV-D8 antiserum bind to the HAdV-D8-like fiber of HAdV-D53 and block infectivity by interfering with virus/primary cellular receptor interaction.

**Table 4:** Neutralization of HAdV-D53 with hyper immune serum.

Antiserum	HAdV-D53	HAdV-D8	HAdV-D22	HAdV-D37
αHAdV-D8	1/128	1/1024	<1/8	<1/8
αHAdV-D22	1/256	<1/8	1/128	<1/8
αHAdV-D37	<1/8	<1/8	<1/8	1/4096

Phylogenetic analysis of the complete genomic sequence of HAdV-D53 showed similar genetic distances to the other available HAdV-D types (6.1% to 9.3% nucleic acid sequence divergence) as observed between other prototypes of species HAdV-D (6.0% to 9.5%) (Fig. 5). This supports the idea that HAdV-D53 is the prototype of a new genotype. Therefore, phylogeny deduced from complete genomic sequence data supports that HAdV-D53 is a new prototype. However, HAdV-D53 is a recombinant virus and its genome is not of monophyletic origin. For most parts of its genome the ancestors of its sequence (HAdV-D8, -D22, -D37) could be identified by bootscan analysis and confirmed by building phylogenetic trees of the corresponding sequence stretches. For example, L1 and L2 of the neutralization determinant ε are highly variable and evolved rapidly by immune escape mechanisms. L1 and L2 of HAdV-D53 were (except for a single point mutation) identical to HAdV-D22 suggesting a recent recombination event in the phylogeny of HAdV-D53. However, bootscan analysis suggested that several regions of HAdV-D53 (IX, IVa2, DNA polymerase, pTP, pVII, protein V, and 18.4K) were dissimilar to all known adenoviruses. Construction of phylogenetic trees supported that these parts of the genome are either from an undiscovered adenovirus or a known yet unsequenced HAdV-D isolate. However, these genome regions are well conserved in HAdV-D and thus led to low, non significant bootstrap values (see Fig. 5 polymerase, protein V and pVII). Additional whole genome sequencing studies of adenovirus prototypes may elucidate whether some of these parts of the HAdV-D53 genome are also derived from recombination events. Interestingly, protein V, a minor capsid protein, was significantly smaller than the homologous proteins of all other members of HAdV-D (e.g. 297 aa vs. 334 aa in HAdV-D46). Moreover, pVII also contained several deletions, nevertheless phylogenetic trees clearly supported clustering of HAdV-D53 protein V and pVII with species HAdV-D in spite of these deletions.

**Figure 6:**
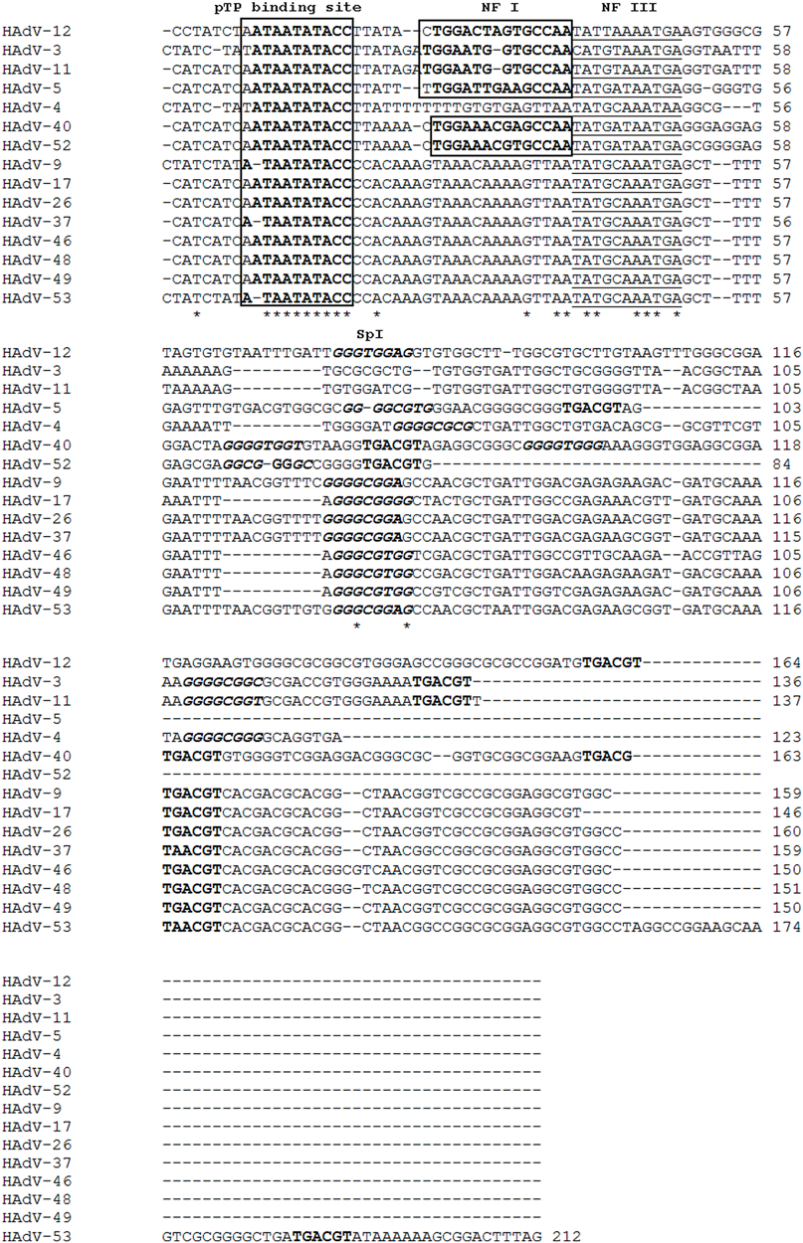
Analysis of the HAdV-D53 inverted terminal repeat (ITR). NF I, NF III, SpI, and pTP binding motifs are marked. The ATF binding site is TGACGT.

The 5′-ITR sequence contains highly conserved critical motifs that are required for adenovirus replication [37]. These motifs include the canonical ‘core origin,’ defined as the minimal DNA requirement for the initiation of replication, binding the terminal protein-DNA polymerase complex [38], and several host transcription factor binding sequences which are required for efficient adenovirus replication [39], [40]. For example, it has been shown that Oct-1 binds to the NF III motif to stimulate transcription by 6–8 fold [41]. Within most HAdV species, both NF I and NF III binding sites are conserved except for species HAdV-E as seen in HAdV-E4 and HAdV-E4 vaccine strain [42], [43] and simian AdV-21 (SAdV-B21), SAdV-E22 through E25 (unpublished observations) which lack the NF I binding site. Significantly, HAdV-D53 is also missing the NF I motif, like the other members of the sequenced HAdV-D types (Fig. 6). Previous annotations of the sequenced HAdV-D members do not remark upon this absence of the NF I. Perhaps this absence of an NF I site is an indication of a different evolutionary line of origin for species HAdV-D, as opposed to the other HAdVs with both NF I and NF III motifs. The latter half of the ITR contains motifs for binding Sp1 (GGG GGT GG) and ATF (TGA CGT). These motifs are also reported to contribute to the efficiency of viral DNA replication [44]. While the Sp1 motif seems to be less conserved (GGG CGg/t gg), they are similar for HAdV-D types. The ATF motif is conserved and present in HAdV-D (Fig. 6).

As new strains of adenoviruses appear and are isolated, usually with an accompanying pathology, initial attempts at understanding the clinical relevance involves characterizing the isolate with respect to structural features. These include the traditional serological methods and reagents. However, in some cases the isolates are difficult to culture and/or the reagents are not readily available. In the past, the isolate is either characterized as much as possible or archived in a laboratory as an unculturable, yet interesting isolate. Today, when an interesting adenovirus isolate arises, full-genome sequencing, phylogenetic analysis, and other state-of-the-art methodology and technology provide alternatives to these limitations. As a recent example, when HAdV-G52 was discovered, it was found that the virus grew too slowly in tissue culture to be ‘properly’ serotyped. This and the lack of readily available serotyping reagents limited a ‘traditional’ characterization. However, phylogenetic analysis, only made possible through whole genome sequencing, demonstrated that it was a novel adenovirus isolate that was quite divergent from all other species of human adenoviruses [6]. Similarly, if serology and limited sequence analysis, e.g., limited hexon, penton and fiber data, were the only tools that we had available for the original characterization of this proposed HAdV-D53, the reported original conclusion in regards to HAdV-D53, that it is a variant of HAdV-D22 albeit with minor genetic modifications in the penton and fiber genes [19], would have been and remained incorrect. In order to conclusively characterize a suspected novel adenovirus, whole genome sequencing and bioinformatics analysis of the resultant and complete reference primary nucleotide sequence should be performed.

The fact that the genes associated with serum neutralization are from known viruses raises a central question, “What are the criteria for defining and naming a new “type” of adenovirus?” Although serology has been crucial in the pre-genomic era, it can not be used as the gold-standard for the typing of novel adenoviruses that will be sequenced and characterized in the future. If serology was the only tool that we had in our typing toolbox, we would not have determined that HAdV-D53 was due to several recombinations of known and perhaps unknown adenoviruses. In the past, the “serotype” designation was used to distinguish different and separate adenoviruses. However, due to the fact that there are about 200 known adenovirus types, this approach is impractical. Moreover, the neutralization of recombinants such as HAdV-B16, and -D53 would yield inconclusive data. Full-genome sequencing and bioinformatic analyses should be the primary methods used when proclaiming novel adenovirus genotypes as it is quicker and a less cumbersome alternative for adenovirus typing, especially given the cost-effective technology to obtain genome sequences rapidly and the growing array of bioinformatics tools, along with the growing adenovirus database.

We propose using “genotype” rather than “serotype” as a means for identifying, characterizing and differentiating adenoviruses, based on genome sequence analyses. This fits into the currently accepted classification of adenovirus “genome types,” in which substrains of adenoviruses are designated by lower case alphabetic designations in addition to their primary designation, e.g., HAdV-7a, b, c…, if their restriction enzyme digestion patterns differ from the reference prototype genome, “HAdV-7p.”

Recently, partial genome sequences from HAdV-D strains causing EKC outbreaks in Japan were published [35], [36]. These were almost identical to HAdV-D53 (including the intrahexon recombination sites) suggesting that HAdV-D53 has already spread around the globe as an emerging EKC agent, reflecting the epidemiology of a globally connected population and a newly emergent pathogen.

## Materials and Methods

### Ethics Statement

The animals involved in this study were procured, maintained, and used in accordance with the Laboratory Animal Welfare Act of 1966, as amended, and NIH 80-23, Guide for the Care and Use of Laboratory Animals, National Research Council.

### Nucleotide sequence accession numbers

The HAdV-D53 genome and annotation have been deposited in GenBank prior to manuscript submission; accession number FJ169625. The following HAdV genomes (GenBank accession numbers) were used: HAdV-A12 (AC_000005), HAdV-B7 (AY594255), HAdV-D8 (AB110079), HAdV-B11 (AY163756), HAdV-C5 (AC_000008), HAdV-E4 (AY599837), HAdV-D49 (DQ393829), HAdV-D53 (FJ169625), HAdV-D9 (AJ854486), HAdV-B16 (AY601636), HAdV-D17 (AC_000006), HAdV-D19 (ER121005), HAdV-D22 (FJ404771) HAdV-D26 (EF153474), HAdV-D37 (DQ900900), HAdV-D46 (AY875648), HAdV-D48 (EF153473), HAdV-D22 (unpublished genome sequence), HAdV-D8 (published partial sequences (AB110079) and unpublished whole genome sequence).

### Amplification of the HAdV-D53 genome

To amplify regions of HAdV-D53 flanking the sequences described by Engelmann et al. [19], we designed primers based on conserved adenovirus sequences of types in HAdV-D. All amplicons were then sequenced using primer walking.

### Viruses, cells and neutralization test

Viral neutralization assays were run as previously described [45]. Rabbit antisera to prototype strains were standardized in cross-neutralization tests against adenovirus prototype viruses 1–49. Prototype viruses were from archives maintained at the State of California, Department of Public Health, Viral and Rickettsial Disease Laboratory.

### Nucleic Acid Isolation

Viral DNA was extracted from tissue culture and processed stool samples using the MagNA Pure LC DNA Isolation Kit I (Roche, Indianapolis, IN) according to the manufacturers' recommendations for the MagNA Pure LC automated nucleic acid extraction system.

### Bioinformatics

Percent idenitities for HAdV-53 genes/proteins. The global alignment were performed using the EMBOSS [46] needle program. The proteins and genes of HAdV-53 were compared to homologs in other HAdV-D genomes. In cases were a genome lacked sufficient annotation, genes and proteins were found manually using the Artemis [47] annotation program. The percent identities for the proteins (Table 2) of the HAdV-D sequences were obtained via BLASTP [48]. The percent identities for the nucleotide sequences (Table 2) that code for these proteins were determined using a BioJava [49] implementation of a Needleman-Wunsch algorithm.

Recombination analysis of hexon genes (Figure 3). Hexons genes from the HAdV-D genomes were aligned using ClustalW [50] alignment option available in the MEGA 4 program [51]. The default gap opening and gap extension penalties were used (15.0 and 6.66). SimPlot [20] software was used to complete a bootscan analysis of the aligned hexon genes of the available HAdV-D genomes. The default settings for window size, a step size, replicates used, gap stripping, distance model, and tree model were, respectively, 200, 20, 100, “on”, “Kimura”, and “Neighbor Joining”. The HAdV-53 hexon was chosen as the reference sequence for the analysis.

Recombination analysis of HAdV-D whole genomes (Figure 2). The available HAdV-D genomes were aligned using the MAFFT [52] alignment method which is available through a web interface at http://www.ebi.ac.uk/Tools/mafft/. The default parameters for gap open penalty, gap extension penalty, and perform fft were used (1.53, 0.12, “localpair”).

Simplot [20] software was used to complete a bootscan analysis of the aligned HAdV-D genomes. The default parameters for window size and step size were altered (1000, 200). All other default parameters were left unchanged.

### Recombination Analysis

Two groups of hexon coding nucleotide sequences were analyzed for recombination events. The first group consisted of the hexon genes of the human adenovirus D species (HAdV-D8, -D9, -D17, -D22, -D26, -D37, -D46, -D48, -D49, -D53). This group is referred to as the HAdV-D53 hexon group. The second group consisted of hexon genes from HAdV-B16, -C5, -E4, -B7, -B11, and -C2. The following accession numbers were used for the hexon recombination analyses. HAdV-A12 (AC_000005), HAdV-B7 (AY594255), HAdV-B11 (AY163756), HAdV-C5 (AC_000008), HAdV-E4 (AY599837), HAdV-D49 (DQ393829), HAdV-D22, (AB330103), HAdV-D53 (FJ169625), HAdV-D9 (AJ854486), HAdV-16/B1 (AY601636), HAdV-D17 (AC_000006), HAdV-D26 (EF153474), HAdV-D37 (DQ900900), HAdV-D46 (AY875648), HAdV-D48 (EF153473). The two groups of sequences were aligned using the ClustalW [50] alignment option available in the MEGA 4 program [51]. The default gap opening and gap extension penalties were used. Those penalties were 15.0 and 6.66 respectively.

Two different programs were used to analyze the two alignments for recombination events. The first program is SimPlot [20]. The bootscan option of SimPlot was used to analyze the alignments. The default settings were used. These included a window size = 200, a step size = 20, replicates used = 100, gap stripping = “on”, distance model = “Kimura”, tree model = “Neighbor Joining”. The HAdV-D53 hexon was chosen as the reference sequence HAdV-D53 hexon group. HAdV-D16's hexon was chosen as the reference in the HAdV-16 hexon group.

The second program is the Recombination Detection Program (RDP) [53]. This program uses several different algorithms (including bootscanning) to determine the presence of recombination events. 1 of the “general recombination detection options” was changed so that the program would recognize that the sequences in the alignment were linear and not circular. No other default options were changed.

### Phylogenetic analysis of HAdV-D53

DNA polymerase, penton base (β-determinant), pVII, protein V, L1 and L2 of the hexon, and fiber knob (γ-determinant) nucleotide sequences were compared by sequential pairwise alignment with the Clustal Algorithm implemented in the BioEdit software package (version 6.0.5) and adjusted manually to conform to the optimized alignment of deduced amino acid sequences. Phylogenetic relationships were inferred from the aligned nucleic acid as well as from the amino acid sequences by the neighbour-joining method implemented in the programs DNAdist and Neighbor integrated in the MEGA software package (version 3.1) using the Kimura two-parameter substitution model and a transition/transversion ratio of 10. Support for specific tree topologies was estimated by bootstrap analysis with 1000 pseudoreplicate data sets.

### *In vivo* model of adenovirus keratitis

Eight to 12 week old C57BL/6J mice (stock # 000664) were purchased from Jackson Laboratory (Bar Harbor, ME). Animal housing and care were in accordance with Animal Care and Use Committee guidelines. Mice were anesthetized for virus infection by intramuscular injection of ketamine (85 mg/kg) and xylazine (14 mg/kg) and later euthanized by CO_2_ inhalation. For infection, 1 microliter of virus-free dialysis buffer, cesium chloride gradient purified HAdV-D22, or purified HAdV-D53 (10^4^ tissue culture infectious dose) was injected in the central corneal stroma as previously described [21]. After euthanasia, corneas were removed and fixed in 10% neutral buffered formalin, embedded in paraffin, and sections cut at 5 µ thick prior to staining. For ELISA, corneas were harvested at indicated time points and homogenized using phosphate buffered saline (PBS) with protease inhibitors, and the reactions performed as per manufacturer's instructions (R&D Systems, Minneapolis, MN). ELISA plates were analyzed on a microplate reader (Molecular Devices, Sunnyvale, CA) with limits of detection of <2 pg/mL for CXCL1 and <1.5 pg/mL for CXCL2. Flow cytometry was performed as described by Carr and coworkers [54]. Corneas were dissected at indicated time points, and digested with 1 mg/ml collagenase type I (Sigma, St. Louis, MO). Non-specific binding was blocked by anti-mouse Fc (BD Pharmingen, San Diego, CA) and 5% normal rat serum (Jackson Immuno Research, West Grove, PA). Cells were labeled with FITC-conjugated anti-mouse F4/80 (clone CI:A3-1), phycoerythrin-Cy5-conjugated anti-CD45 (clone 30-F11), and PE-conjugated anti-mouse Gr-1 (clone RB6-8C5) (all from BD Biosciences, San Jose, CA). and incubated in the dark on ice for 30 min, washed 3× with PBS/1% BSA, resuspended in PBS containing 1% paraformaldehyde, and incubated overnight. CountBright absolute counting beads (Invitrogen, Eugene, OR) were added (21,600 beads/sample), cell suspensions gated on CD45^high^ labeled cells, and the numbers of each cell type determined at this gate setting. A second gate was established to count the number of beads that passed through during each run (300 sec). The absolute number of cells per cornea was determined by calculating the number of input beads/21,600× number of cells in the CD45^high^-gated sample.
